# Inhaling Bad Air

**Published:** 1875-02

**Authors:** 


INHALING BAD AIR.—It is important to know how localities in which the air is rendered unfit for respiration by reason of the existence of smoke or gas or dust or soot, can be entered without danger of suffocation. Firemen are constantly thus exposed, as are workers in chemical laboratories, miners, stokers, iron-puddlers, and others. In Great Britain, an armor is in use, having a metal reservoir of compressed air at the back, from which a tube extends to the mouth. This allows the explorer in deadly localities to exist for a limited period, depending upon the stored-up air for support. But the system is awkward, and any injury to the apparatus leaves the wearer at the mercy of the adverse conditions. A better arrangement is found in a closely-fitting mask, having glasses for the eyes, and with a padding of two thicknesses of felt with a layer of sponge between them, the whole saturated with water, and so formed as to cover the mouth and nose. A mask of this kind, having curtains attached for the protection of the neck, is found to work admirably, and to give immunity to the wearer for a period varying from a quarter of an hour to an hour in an atmosphere filled with smoke or choked with sulphuretted and other dangerous gases. We have seen a party of men who were provided with simple fixtures of this sort, fight and overcome a prairie fire, before which unprotected persons were compelled to fly for dear life, so dense and suffocating was the smoke. The wet fabric completely strains the air of all impurities, and leaves it in a fit state to sustain life.

In practice we are doing the same thing every hour of our lives, although we rarely think of it. The nose is the moistened strainer, and those who are wise enough to keep their mouths shut amid dust, and gas, and noxious odors, are saved from many dangers. The interior of the nose bristles thickly with hairs, which impede the passage of extraneous substances to the lungs. It is far more important than people imagine that the mouth be kept closed in a bad atmosphere; by which we mean an atmosphere vitiated by unwholesome odors, or by substances suspended in it. In cutlery establishments it is noticed that the silent grinders live about twice as long as the talkative ones. The air of the rooms in which the edge is put on by rapidly-revolving dry grindstones, always holds in suspension a cloud of dust of steel and stone, and the average grinder does not usually live to be over 30 years old. Disease is often inhaled through the mouth which would have been avoided had breathing been carried on by the nose, and it is safe to say that the contagion of diptheria would usually be avoided if only the nasal passages were employed in respiration, as the bacteria-germs would rarely, if ever, reach the necessary position in the throat, through a channel so closely guarded. The horrible and wholly needless habit of snoring can only exist in cases in which breathing through the mouth is habitual, and will be cured as soon as the custom is changed, and the nose is used as nature intended it to be.

Prof. Tyndall has devised a hood, having a mouth-piece filled with cotton, saturated with glycerine, and supplied with lime and charcoal. The value of the glycerine rests in the fact that it is not likely to dry up, as it evaporates very slowly; while the charcoal and lime neutralize the noxious gases. The truth is that whatever perfectly strains the air answers the purpose, and more simple the device, the more perfectly will it subserve the purpose.

